# Surgeon and Care Team Network Measures and Timely Breast Cancer Treatment

**DOI:** 10.1001/jamanetworkopen.2024.27451

**Published:** 2024-08-29

**Authors:** Ramsey Ash, Bruno T. Scodari, Andrew P. Schaefer, Sarah L. Cornelius, Gabriel A. Brooks, A. James O’Malley, Tracy Onega, Dana C. Verhoeven, Erika L. Moen

**Affiliations:** 1 Program in QUantitative Social Science, Dartmouth College, Hanover, New Hampshire; 2 Department of Biomedical Data Science, Geisel School of Medicine at Dartmouth, Lebanon, New Hampshire; 3 The Dartmouth Institute for Health Policy and Clinical Practice, Lebanon, New Hampshire; 4 Dartmouth Cancer Center, Dartmouth Hitchcock Medical Center, Lebanon, New Hampshire; 5 Department of Medicine, Geisel School of Medicine at Dartmouth, Lebanon, New Hampshire; 6 Department of Population Health Sciences, University of Utah, Salt Lake City; 7 Huntsman Cancer Institute, University of Utah, Salt Lake City; 8 Department of Health Services Research & Administration, University of Nebraska Medical Center College of Public Health, Omaha

## Abstract

**Importance:**

Cancer treatment delay is a recognized marker of worse outcomes. Timely treatment may be associated with physician patient-sharing network characteristics, yet this remains understudied.

**Objective:**

To examine the associations of surgeon and care team patient-sharing network measures with breast cancer treatment delay.

**Design, Setting, and Participants:**

This cross-sectional study of Medicare claims in a US population-based setting was conducted from 2017 to 2020. Eligible participants included patients with breast cancer who received surgery and the subset who went on to receive adjuvant therapy. Patient-sharing networks were constructed for treating physicians. Data were analyzed from September 2023 to February 2024.

**Exposures:**

Surgeon linchpin score (a measure of local uniqueness or scarcity) and care density (a measure of physician team familiarity) were assessed. Surgeons were considered linchpins if their linchpin score was in the top 15%. The care density of a patient’s physician team was calculated on preoperative teams for surgically-treated patients and postoperative teams for adjuvant therapy-receiving patients.

**Main Outcomes and Measures:**

The primary outcomes were surgical and adjuvant delay, which were defined as greater than 60 days between biopsy and surgery and greater than 60 days between surgery and adjuvant therapy, respectively.

**Results:**

The study cohort included 56 433 patients (18 004 aged 70-74 years [31.9%]) who were mostly from urban areas (44 931 patients [79.6%]). Among these patients, 8009 (14.2%) experienced surgical delay. Linchpin surgeon status (locally unique surgeon) was not statistically associated with surgical delay; however, patients with high preoperative care density (ie, high team familiarity) had lower odds of surgical delay compared with those with low preoperative care density (odds ratio [OR], 0.58; 95% CI, 0.53-0.63). Of the 29 458 patients who received adjuvant therapy after surgery, 5700 (19.3%) experienced adjuvant delay. Patients with a linchpin surgeon had greater odds of adjuvant delay compared with those with a nonlinchpin surgeon (OR, 1.30; 95% CI, 1.13-1.49). Compared with those with low postoperative care density, there were lower odds of adjuvant delay for patients with high postoperative care density (OR, 0.77; 95% CI, 0.69-0.87) and medium postoperative care density (OR, 0.85; 95% CI, 0.77-0.94).

**Conclusions and Relevance:**

In this cross-sectional study of Medicare claims, network measures capturing physician scarcity and team familiarity were associated with timely treatment. These results may help guide system-level interventions to reduce cancer treatment delays.

## Key Points

**Question:**

Are surgeon and care team patient-sharing network measures associated with breast cancer treatment delays?

**Findings:**

This cross-sectional study of 56 433 Medicare beneficiaries with breast cancer found that patients with a surgeon representing a unique or scarce specialty among their network had significantly greater odds of delayed adjuvant treatment compared with those without such a surgeon. Additionally, patients whose care team had a greater extent of patient sharing had significantly lower odds of surgical and adjuvant treatment delay compared with those with a lower extent of patient sharing.

**Meaning:**

These findings suggest that network measures capturing surgeons and team familiarity may help guide efforts to reduce treatment delays.

## Teaser

This cross-sectional study assesses associations of surgeon and care team patient-sharing networks with access to timely treatment among patients with breast cancer.

## Introduction

Delays in cancer treatment are often associated with worse survival.^1,2^ A recent meta-analysis^2^ found that a 4-week delay in cancer treatment was associated with increased mortality for all treatment modalities (surgery, chemotherapy, and radiotherapy) and across 7 cancer types. Treatment delays across phases of care are consequential, with most studies focusing on the intervals between biopsy and surgery and surgery and adjuvant therapy.^2,3^ Because treatment delay is a modifiable marker of worse outcomes, understanding the system-level factors associated with treatment delay is critical for informing strategies to improve the quality of cancer care delivery.

The structure of physician networks may provide insight into the coordination and timeliness of cancer treatment initiation. Physician networks can be inferred using claims data by connecting those who shared patients.^4,5^ Recent advancements in network analysis have leveraged these patient-sharing networks to develop several measures that capture elements of care coordination. The linchpin score represents a physician-level measure of local uniqueness or scarcity of that specialty type within the patient-sharing network.^6^ For instance, a surgeon would have a high linchpin score when few or none of the physicians they share patients with are connected to another surgeon. Prior research^7,8,9^ found that patients treated by linchpin oncologists were more likely to be socioeconomically disadvantaged, rural-residing, and often had worse survival. Care density reflects the extent of patient-sharing among a patient’s care team. Patients whose treating physicians share many patients will have high care density. Higher care density has been associated with lower geographic dispersion of cancer care and lower costs of survivorship care.^10,11^ Higher care density has also been associated with lower odds of hospital readmission for patients with diabetes^12^ and lower costs of care for patients with diabetes and congestive heart failure.^13^ Despite the important role of coordination among multidisciplinary clinicians in cancer treatment planning, few studies have used network analysis to determine which characteristics of patient-sharing networks facilitate timely cancer treatment.^14,15^

The objective of this study was to examine associations of patient-sharing network measures with timely treatment for patients with breast cancer. We focused on breast cancer due to existing guidelines surrounding the timeliness of surgical and adjuvant therapy initiation and evidence that timely treatment improves survival.^16,17,18,19,20,21,22,23^ Because surgeons play a critical role in coordinating treatment for patients with breast cancer during the preoperative and postoperative phases, we calculated linchpin score for surgeons and care density for preoperative and postoperative teams.^24,25^ Our overarching hypothesis was that patients treated by a linchpin surgeon and physician teams with low care density would be more likely to experience delays in treatment.

## Methods

### Data and Approvals

This cross-sectional study was approved by the Dartmouth College institutional review board with a waiver of informed consent and followed the Strengthening the Reporting of Observational Studies in Epidemiology (STROBE) reporting guideline. We used 100% fee-for-service Medicare claims from 2017 to 2020 to identify patients with incident breast cancer and their treating physicians. The data are not publicly available due to a data use agreement with the Centers for Medicare & Medicaid Services.

### Study Cohort

We identified female patients with a breast biopsy followed by 2 breast cancer diagnosis codes within 12 months (eTable 1 in Supplement 1).^26^ To enrich for patients with incident cancer, we excluded patients with a cancer diagnosis code in the 12 months prior to biopsy. We also excluded patients who were younger than 66 years or older than 99 years at the time of biopsy, were not continuously enrolled in Medicare Parts A and B in the 12 months prior to and following biopsy, received multiple cancer diagnoses, or had a missing or non-US zip code. Furthermore, we excluded those who received a diagnosis of metastatic disease within 90 days of biopsy, treated with neoadjuvant chemotherapy or radiotherapy, or received reconstructive surgery on the day of surgery. We applied these restrictions due to prior research that found associations of these characteristics with longer times to surgical and adjuvant therapy initiation for patients with breast cancer.^27,28,29,30,31^

### Network Assembly

We linked physicians who provided care to patients in the study cohort in the 3 months prior to and/or 12 months following biopsy using carrier claims. We constructed a weighted patient-sharing network where physicians were connected if they shared at least 3 patients. Each physician in the network was assigned a specialty according to the following classifications: medical oncologist, radiation oncologist, surgeon, or other. To reduce noise, we restricted the network to physicians who provided care to at least 5 patients.^7^

### Treatment Cohorts

We identified the first cancer-directed surgery and the first adjuvant chemotherapy or radiotherapy (if applicable) for each patient in the study cohort by requiring a procedure code associated with a physician included in the network and of the expected specialty (eg, surgeon for surgery) (eTable 2 and eTable 3 in Supplement 1). These observations were partitioned into separate surgery and adjuvant therapy treatment cohorts, with the adjuvant therapy cohort being a subset of the surgery cohort.^3^

### Study Variables

#### Patient-Level Variables

Patient age and race were determined using the Centers for Medicare & Medicaid Services Master Beneficiary Summary file. Race and ethnicity was considered a social construct and included due to its hypothesized associations with the exposure and outcome variables.^32,33,34^ Race and ethnicity categories included Asian, Black, Hispanic, North American Native, White, unknown, and other (defined as any race or ethnicity not otherwise specified). We assigned patients an economic deprivation index measure based on the percentage of persons below the poverty line for their residential zip code and categorizing this value into bins: very low (<5.00%), low (5.00%-9.99%), medium (10.00%-14.99%), high (15.00%-19.99%), and very high (≥20.00%).^35^ Patient rurality was ascribed using residential zip codes according to a 4-tier classification of rural-urban commuting area codes (urban, large rural, small rural, or isolated).^36^ The number of Charlson comorbidities was assigned by the Klabunde method using claims from the 12 months preceding biopsy and binned as 0, 1, and 2 or more comorbidities.^37^ National Cancer Institute (NCI) affiliation was based on whether the patient’s first surgery occurred at an NCI-designated cancer center.

#### Physician-Level Variables

Physician gender and specialty were obtained from the National Plan and Provider Enumeration System National Downloadable File (eTable 3 in Supplement 1). Patient volume was estimated by tallying the number of patients in the study cohort a physician provided care to over the study period. Physician rurality was assigned with the same rural-urban commuting area classification using their modal zip code. A measure of oncologist supply was calculated as the ratio of medical, radiation, and surgical oncologists (herein referred to as oncologists) to persons older than 65 years within each hospital referral region (HRR) and assigned to physicians based on their modal HRR. Lastly, we calculated 2 physician-level measures of degree centrality to reflect the extent to which an oncologist’s patient-sharing ties to other oncologists were local vs regional. The first was within-hospital service area (HSA) degree, which was calculated as the number of unique patient-sharing ties to oncologists with the same primary HSA, and the second was between-HSA degree, which was calculated as the number of unique patient-sharing ties to oncologists with a different primary HSA. We binned all continuous physician-level attributes into tertiles.

#### Encounter-Level Variables

Surgery, chemotherapy, and radiotherapy procedures were identified for each patient using procedure codes and physician specialty information. The number of clinical encounters between a patient’s biopsy and their first surgery was tallied, as was the number of clinical encounters between a patient’s last surgery and their first adjuvant therapy. We included these variables as covariates to account for any additional services (eg, imaging) provided to the patient, which have been associated with surgical delay, as well as other services related to unmeasured aspects of disease complexity that may contribute to delay.^3,23,28^

### Exposure Variables

Two exposure variables were defined separately for the surgery and adjuvant therapy cohorts. The first exposure was the linchpin score of a patient’s surgeon, a measure of surgeon scarcity. Linchpin score is calculated for a given surgeon by summing the weights of patient-sharing ties to peers who lacked ties to another surgeon and dividing by the sum of all ties^6^ (Figure, A). A linchpin surgeon was defined as any surgeon with a linchpin score within the top 15%. We selected this threshold based on prior work that associated this definition of a linchpin oncologist with increased mortality among patients with cancer.^8^ The second exposure was the care density of a patient’s team of physicians, a measure of team familiarity. A patient’s team was defined as the set of physicians they have visits with during a given time period. The numerator of care density is the sum of shared patients among each pair of physicians on a patient’s team, and the denominator is the total number of physician pairs on the team (Figure, B).^13^ Care density was calculated on the preoperative team for the surgery cohort and the postoperative team for the adjuvant therapy cohort.

**Figure.**
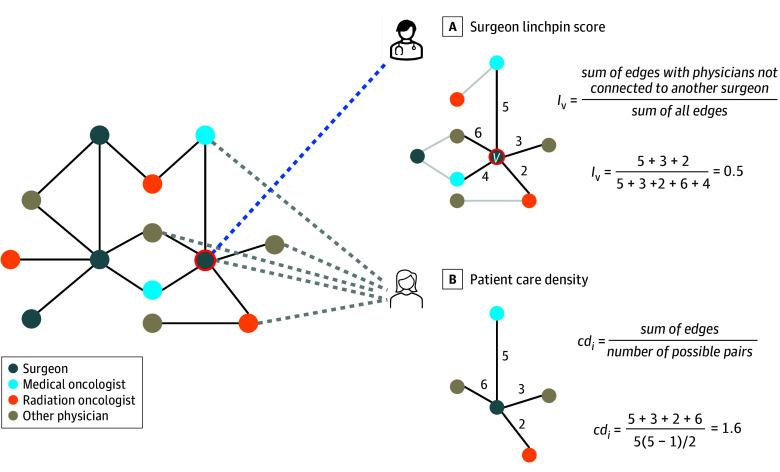
Illustration of the Network Measures of Interest A, Focal surgeon *v* is connected to 3 physicians who lack ties to another surgeon in the patient-sharing network, and their edge weights sum to 10 (5 + 3 + 2). The sum of all edge weights is 20 (5 + 3 + 2 + 6 + 4). Therefore, the linchpin score (*I*) of surgeon *v* is calculated as 10 / 20 = 0.5. B, Over the course of their treatment, patient *i* received care from 5 physicians, of whom have 10 possible ties (5 × [5 - 1] / 2 = 10). The existing ties between this set of physicians have edge weights that sum to 16 (5 + 3 + 2 + 6). Therefore, the care density (*cd*) for patient *i* is calculated as 16 / 10 = 1.6.

#### Surgery Cohort

In the surgery cohort, the first exposure variable was the linchpin status of a patient’s operating surgeon. The second exposure variable was a patient’s preoperative care density (tertiles), which was defined as the care density computed for all physicians who provided care to the patient during the interval between 3 months prior to biopsy and surgery. Physicians who provided diagnostic or consultative services during this period, such as second or third opinions, would be included in this calculation. For patients who only received care from their surgeon during the preoperative period, we defined a fourth level of preoperative care density called sole clinician because their preoperative care density was mathematically undefined (due to 0 division).

#### Adjuvant Therapy Cohort

In the adjuvant therapy cohort, the first exposure variable was the linchpin status of a patient’s surgeon. The second exposure variable was a patient’s postoperative care density (tertiles), which was defined as the care density computed for oncologists who provided care to the patient during the interval from surgery to adjuvant therapy.

### Outcome Variables

The outcomes of interest were surgical and adjuvant delay, which were defined as greater than 60 days between biopsy-to-surgery and surgery-to-adjuvant therapy intervals, respectively. We selected a cutoff of greater than 60 days due to prior research that found associations of adverse patient outcomes with surgical and adjuvant delays greater than approximately 60 days.^17,20,21,22^ In sensitivity analysis, we considered an alternate cutoff of greater than 90 days due to its use as a less conservative definition for delay in other studies.^3,16,18,19,23^

### Statistical Analysis

We used bivariate analysis to explore the associations of patient characteristics with the linchpin status of their surgeon and care density measures. Similarly, we used bivariate analysis to explore the surgeon characteristics associated with linchpin status. In the surgery cohort, we used hierarchical logistic regression to investigate the associations of the linchpin status of a patient’s surgeon and the patient’s preoperative care density with surgical delay. In the adjuvant therapy cohort, we used similar models to investigate the associations of the linchpin status of a patient’s surgeon and the patient’s postoperative care density with adjuvant delay. For each cohort, we included both exposure variables in the same model and adjusted for all encounter-, patient-, and physician-level study variables to control for potential confounding. We also included cross-classified random intercepts for the physician (using the National Provider Identifier) and beneficiary HRR to account for within-cluster correlation. We reported odds ratios (ORs), 95% CIs, and *P* values. Statistical significance was considered a 2-sided *P* < .05. Analyses were conducted using R statistical software version 4.3.1 (R Project for Statistical Computing) and took place from September 2023 to February 2024.

## Results

We identified 56 433 patients with incident breast cancer (18 004 aged 70-74 years [31.9%]; 3437 Black [6.1%]; 50 200 White [89.0%]; 1634 other or unknown race [2.9%]) with breast cancer who received surgery (surgery cohort), of whom 44 931 (79.6%) lived in urban areas. Of these patients, 29 458 (52.2%) received adjuvant chemotherapy or radiotherapy and were included in the adjuvant therapy cohort (eFigure in Supplement 1).

### Patient Characteristics Associated With Exposure Variables

Of the patients in the surgery cohort, 8874 (15.7%) received surgery from a linchpin surgeon. Patients who were treated by a linchpin surgeon were more likely to be older (χ^2^_4_ = 19.0; *P* < .001), more likely to live in a rural (vs urban) area (χ^2^_3_ = 395.1; *P* < .001), more likely to live in an economically deprived area (χ^2^_4_ = 101.2; *P* < .001), more likely to have greater comorbidity (χ^2^_2_ = 70.3; *P* < .001), and more likely to be treated at a center unaffiliated with the NCI (χ^2^_1_ = 828.3; *P* < .001) (Table 1).

**Table 1.** Bivariate Associations of Patient Attributes With Receipt of Surgery From a Linchpin Surgeon

Characteristic	Patients, No. (%) (N = 56 433)		
	Received surgery from a nonlinchpin surgeon (n = 47 559)	Received surgery from a linchpin surgeon (n = 8874)	*P* value^a^
Age at diagnosis, y			
66-69	12 076 (25.4)	2115 (23.8)	<.001
70-74	15 169 (31.9)	2835 (31.9)	
75-79	10 679 (22.5)	1973 (22.2)	
80-84	5990 (12.6)	1233 (13.9)	
≥85	3645 (7.7)	718 (8.1)	
Race and ethnicity			
Asian	565 (1.2)	94 (1.1)	<.001
Black	2822 (5.9)	615 (6.9)	
Hispanic	275 (0.6)	53 (0.6)	
North American Native	141 (0.3)	34 (0.4)	
Other^b^	702 (1.5)	105 (1.2)	
Unknown	721 (1.5)	106 (1.2)	
White	42 333 (89.0)	7867 (88.7)	
Comorbidities, No.			
0	26 087 (54.9)	4482 (50.5)	<.001
1	10 791 (22.7)	2078 (23.4)	
≥2	10 681 (22.5)	2314 (26.1)	
Economic Deprivation Index			
Very low	12 952 (27.2)	2073 (23.4)	<.001
Low	22 816 (48.0)	4234 (47.7)	
Medium	7993 (16.8)	1674 (18.9)	
High	2329 (4.9)	567 (6.4)	
Very high	1469 (3.1)	326 (3.7)	
National Cancer Institute affiliation			
No	41 107 (86.4)	8626 (97.2)	<.001
Yes	6452 (13.6)	248 (2.8)	
Rurality			
Isolated	1887 (4.0)	506 (5.7)	<.001
Small rural	2389 (5.0)	744 (8.4)	
Large rural	4738 (10.0)	1238 (14.0)	
Urban	38 545 (81.0)	6386 (72.0)	

^a^ *P* values calculated using Pearson χ^2^ test for independence.

^b^ Other included any race or ethnicity not otherwise specified.

Of the 29 458 patients in the adjuvant therapy cohort (10 160 aged 70-74 years [34.5%]; 1787 Black [6.1%]; 26 162 White [88.8%]; 904 other or unknown race [3.1%]), 4528 (15.4%) received surgery from a linchpin surgeon (eTable 4 in Supplement 1). Patient characteristics and their associations with both preoperative and postoperative care density tertiles are reported in eTable 5 in Supplement 1.

### Characteristics of Linchpin Surgeons

We linked 5631 surgeons to the 56 433 patients in the surgery cohort. Linchpin surgeons were more likely to practice in rural vs urban areas, (χ^2^_3_ = 62.1; *P* < .001) more likely to practice in areas of low (vs medium or high) oncologist supply (χ^2^_2_ = 35.5; *P* < .001), more likely to have low (vs medium or high) patient volume (χ^2^_2_ = 6.7; *P* = .04), and more likely to have fewer ties to physicians within their HSA (ie, a low vs medium or high within-HSA degree; χ^2^_2_ = 64.6; *P* < .001) (Table 2).

**Table 2.** Bivariate Associations of Surgeon Attributes and Linchpin Status

Characteristic	Surgeons, No. (%) (N =5631)		*P* value^a^
	Nonlinchpin surgeon (n = 4746)	Linchpin surgeon (n = 885)	
Gender			
Woman	1967 (41.4)	353 (39.9)	.41
Man	2779 (58.6)	532 (60.1)	
Patient volume			
Low	1450 (30.6)	309 (34.9)	.04
Medium	1559 (32.8)	268 (30.3)	
High	1737 (36.6)	308 (34.8)	
Rurality			
Isolated	35 (0.7)	12 (1.4)	<.001
Small rural	138 (2.9)	59 (6.7)	
Large rural	605 (12.7)	165 (18.6)	
Urban	3968 (83.6)	649 (73.3)	
Oncologist supply			
Low	1368 (28.8)	337 (38.1)	<.001
Medium	1711 (36.1)	308 (34.8)	
High	1667 (35.1)	240 (27.1)	
Within-HSA degree			
Low	1509 (31.8)	368 (41.6)	<.001
Medium	1555 (32.8)	322 (36.4)	
High	1682 (35.4)	195 (22.0)	
Between-HSA degree			
Low	1594 (33.6)	283 (32.0)	.62
Medium	1573 (33.1)	304 (34.4)	
High	1579 (33.3)	298 (33.7)	

Abbreviation: HSA, hospital service area.

^a^ *P* values calculated using Pearson χ^2^ test for independence.

### Treatment Delays and Associations With Patient-Sharing Network Measures

In the surgery cohort, 8009 patients (14.2%) experienced surgical delay. Receipt of surgery from a linchpin surgeon was not associated with surgical delay (OR, 0.91, 95% CI, 0.81-1.01). However, compared with those with low preoperative care density, there were lower odds of surgical delay for patients with high preoperative care density (OR, 0.58; 95% CI, 0.53-0.63) and those with a sole clinician during the preoperative period (OR, 0.35; 95% CI, 0.33-0.38) (Table 3 and eTable 6 in Supplement 1). In the adjuvant therapy cohort, among patients who received adjuvant therapy after surgery, 5700 (19.3%) experienced adjuvant delay. Patients whose treating surgeon was a linchpin had increased odds of adjuvant delay compared with those whose surgeon was not a linchpin (OR, 1.30; 95% CI, 1.13-1.49). We also found that compared with those with low postoperative care density, there were lower odds of adjuvant delay for patients with high postoperative care density (OR, 0.77; 95% CI, 0.69-0.87) and medium postoperative care density (OR, 0.85; 95% CI, 0.77-0.94) (Table 3 and eTable 7 in Supplement 1). Our results were largely robust to changing the treatment delay cutoff to greater than 90 days (eTable 8 in Supplement 1).

**Table 3.** Adjusted Associations of Exposures With Treatment Delay, Stratified by Treatment Cohort^a^

Exposure	Surgery cohort		Adjuvant therapy cohort	
	OR (95% CI)	*P* value	OR (95% CI)	*P* value
Linchpin status of surgeon (yes vs no)	0.91 (0.81-1.01)	.08	1.30 (1.13-1.49)	<.001
Preoperative care density				
Medium vs low	0.96 (0.89-1.04)	.33	NA	NA
High vs low	0.58 (0.53-0.63)	<.001	NA	NA
Sole-clinician vs low	0.35 (0.33-0.38)	<.001	NA	NA
Postoperative care density				
Medium vs low	NA	NA	0.85 (0.77-0.94)	.002
High vs low	NA	NA	0.77 (0.69-0.87)	<.001

Abbreviation: NA, not applicable.

^a^ All statistical models incorporate random intercepts for physician National Provider Identifier and beneficiary hospital referral region and control for all patient-, physician-, and encounter-level variables. Please refer to eTables 4-8 in Supplement 1 for full model results.

## Discussion

In this cross-sectional study of Medicare claims, we found that receipt of surgery from a linchpin surgeon was associated with increased odds of adjuvant delay, and that higher care density during the preoperative and postoperative period reduced the odds of surgical and adjuvant delay, respectively. To our knowledge, this is the first study to examine associations of surgeon and care team network measures with timely treatment across the phases of breast cancer care.

Our analyses of surgeon linchpin status build on the relatively limited work that has examined associations of surgeon characteristics with treatment delay.^23^ Breast cancer surgeons play a critical role in coordinating care for surgically treated patients who are then referred to adjuvant treatment, which often involves sequential encounters with multidisciplinary clinicians.^24,25^ Interestingly, receiving treatment from linchpin surgeons was not associated with surgical delay, which may be explained by strong relationships with referring upstream clinicians or lack of access to second opinions or other preoperative services (eg, imaging) in networks where surgeons are scarce. Receiving treatment from linchpin surgeons was associated with increased odds of adjuvant delay. This finding could be due to lower numbers of local (within-HSA) ties with other cancer specialists (Table 1), needing to coordinate across health care systems, or a function of practicing in areas with fewer resources for streamlining care (eg, nurse navigators). Although our models adjusted for oncologist supply—a surrogate for oncologist availability—it is possible that these delays are partially due to other barriers in accessing oncology specialty care (eg, lack of transportation).

Likewise, our findings concerning care density are consistent with previous research. A prior study^15^ found that patients treated by physicians in highly connected networks were less likely to experience surgical delay. Care density enables a deeper investigation into how relationships between specific sets of multidisciplinary cancer clinicians relate to the quality of care, and we found that patients with higher preoperative and postoperative care density had reduced odds of treatment delay. Furthermore, we found that patients with a sole clinician (surgeon) during the postoperative period had reduced odds of surgical delay compared with those with high preoperative care density. We postulate that these patients may not require or have access to multidisciplinary cancer teams, which could result in expedited surgery in lieu of additional evaluation.

Our results have implications for policies aimed at improving timely treatment for patients with cancer. The identification of linchpin surgeons using administrative claims provides an opportunity to assist such physicians and their patients with interventions designed to improve care timeliness and coordination. For example, prior research^38,39^ has shown that patient navigation can significantly reduce the time to treatment for at-risk patients. The associations of patient navigation programs with reducing delays in patients treated by linchpin surgeons could be explored. Additionally, strategies aimed at increasing care coordination could leverage care density to track changes in familiarity and cohesiveness of physician teams. Policies that may encourage higher care density include health care payment and delivery models such as Oncology Medical Homes or Oncology Care Models. Future work could examine associations of care density with additional outcomes in the context of implementing new payment and delivery models.^40,41^

### Limitations

Our study has several limitations. First, the patient-sharing network was constructed based on inferred rather than self-reported relationships, and it did not include all physicians who provided care to patients in the study cohort due to the imposed restrictions. Second, the proposed network measures of linchpin score and care density are dependent on the thresholds used to classify linchpin surgeons and low, medium, and high care-density tertiles. We selected our thresholds based on prior research^8,10,12,13^ that used these specific definitions to associate these network measures with patient outcomes. Third, our study only considered patients with fee-for-service Medicare coverage and nonmetastatic breast cancer; therefore, the reported results may not generalize to all patients with cancer. Fourth, our analysis did not consider those who received neoadjuvant endocrine therapy because this treatment modality is not identifiable in carrier claims. Fifth, the reported associations are prone to unmeasured confounding because we were unable to quantify certain variables in Medicare claims that may distort the exposure-outcome associations (eg, cancer stage or tumor receptor status). Lastly, due to the observational nature of our data and cross-sectional study design, we cannot derive any causal inference or rule out potential reverse causality.

## Conclusions

In this cross-sectional study of Medicare claims, we found that surgeon and care team patient-sharing measures were associated with timely breast cancer treatment. Future research may consider additional network measures or explore how care coordination interventions perform across different network structures. Our study suggests that patient-sharing network measures are meaningful markers of timely cancer care, which may inform strategies aimed at measuring and intervening on physician networks to improve the quality of patient-centered care.

## Notes

Supplement 1. **eTable 1.** *CPT *and *ICD-10* Codes Used to Identify Incident Breast Cancer Patients **eTable 2.** *CPT* and HCPCS Codes Used to Identify Breast Cancer Procedures **eTable 3.** Taxonomy Codes Used to Identify Physician Specialty **eFigure.** Patient Flow Diagram **eTable 4.** Bivariate Associations Between Patient Attributes of Adjuvant Therapy-Receiving Patients and Receipt of Surgery From a Linchpin Surgeon **eTable 5.** Bivariate Associations Between Patient Attributes With Pre- and Postoperative Care Density Tertiles **eTable 6.** Adjusted Associations Between Exposures and Surgical Delay **eTable 7.** Adjusted Associations Between Exposures and Adjuvant Delay **eTable 8.** Adjusted Associations Between Exposures and Treatment Delay When Changing the Treatment Delay Cutoff to >90 Days, Stratified By Treatment Cohort

Supplement 2.
Data Sharing Statement
