# Supplementary material for: Surgeon and Care Team Network Measures and Timely Breast Cancer Treatment
Source: JAMA Netw Open. 2024 Aug 29;7(8):e2427451. doi: 10.1001/jamanetworkopen.2024.27451 (PMC11362867; doi:10.1001/jamanetworkopen.2024.27451)
Supplement: Supplement 1. — eTable 1. CPT and ICD-10 Codes Used to Identify Incident Breast Cancer Patients eTable 2. CPT and HCPCS Codes Used to Identify Breast Cancer Procedures eTable 3. Taxonomy Codes Used to Identify Physician Specialty eFigure. Patient Flow Diagram eTable 4. Bivariate Associations Between Patient Attributes of Adjuvant Therapy-Receiving Patients and Receipt of Surgery From a Linchpin Surgeon eTable 5. Bivariate Associations Between Patient Attributes With Pre- and Postoperative Care Density Tertiles eTable 6. Adjusted Associations Between Exposures and Surgical Delay eTable 7. Adjusted Associations Between Exposures and Adjuvant Delay eTable 8. Adjusted Associations Between Exposures and Treatment Delay When Changing the Treatment Delay Cutoff to >90 Days, Stratified By Treatment Cohort [file jamanetwopen-e2427451-s001.pdf]

Supplemental Online Content

Ash R, Scodari BT, Schaefer AP, et al. Surgeon and care team network measures and timely breast cancer treatment. *JAMA Netw. Open*. 2024;7(8):e2427451.
doi:10.1001/jamanetworkopen.2024.27451

eTable 1. *CPT* and *ICD-10* Codes Used to Identify Incident Breast Cancer Patients
eTable 2. *CPT* and HCPCS Codes Used to Identify Breast Cancer Procedures
eTable 3. Taxonomy Codes Used to Identify Physician Specialty
eFigure. Patient Flow Diagram
eTable 4. Bivariate Associations Between Patient Attributes of Adjuvant Therapy-Receiving Patients and Receipt of Surgery From a Linchpin Surgeon
eTable 5. Bivariate Associations Between Patient Attributes With Pre- and Postoperative Care Density Tertiles
eTable 6. Adjusted Associations Between Exposures and Surgical Delay
eTable 7. Adjusted Associations Between Exposures and Adjuvant Delay
eTable 8. Adjusted Associations Between Exposures and Treatment Delay When Changing the Treatment Delay Cutoff to >90 Days, Stratified By Treatment Cohort

This supplemental material has been provided by the authors to give readers additional information about their work.

© 2024 Ash R et al. *JAMA Network Open*.

eTable 1. *CPT* and *ICD-10* Codes Used to Identify Incident Breast Cancer Patients

Procedure	Codes
Diagnosis[a]	ICD-10: C50.X
Biopsy	CPT: 10021, 10022, 19000, 19001, 19081, 19082, 19083, 19084, 19085, 19086, 19100, 19101, 19120, 19125, 19126, 19281, 19282, 19283, 19284, 19285, 19286, 19287, 19288<br><br>ICD-10: 0HBT0ZX, 0HBT3ZX, 0HBT4ZX, 0HBT7ZX, 0HBT8ZX, 0HBU0ZX, 0HBU3ZX, 0HBU4ZX, 0HBU7ZX, 0HBU8ZX, 0HBV0ZX, 0HBV3ZX, 0HBV4ZX, 0HBV7ZX, 0HBV8ZX

[a] Excludes noninvasive cases.
CPT: Current Procedural Terminology
ICD-10: International Classification of Diseases, Tenth Revision

eTable 2. *CPT* and HCPCS Codes Used to Identify Breast Cancer Procedures

Procedure	Codes
Chemotherapy	CPT: 36640, 51720, 96401, 96405, 96406, 96408, 96410, 96412, 96414, 96420, 96422, 96423, 96425, 96440, 96445, 96450, 96501, 96504, 96505, 96508, 96510, 96511, 96512, 96520, 96524, 96530, 96538, 96540, 96542, 96545, 96549, 96450, 96555<br><br>HCPCS: J0202, J1675, J1930, J1950, J2353, J2354, J2860, J3315, J3316, J7504, J7511, J8527, J8520, J8530, J8560, J8565, J8565, J8600, J8705, J8999, J9000-J9999
Radiation	CPT: 77261, 77262, 77263, 77280, 77281, 77282, 77283, 77284 77285, 77286, 77287, 77289 77290, 77295, 77300, 77301, 77331, 77338, 77306, 77307, 77316, 77317, 77318, 77321, 77332, 77333, 77334, 77336, 77370, 77761, 77762, 77763, 77767, 77768, 77770, 77771, 77772, 77778, 0394T, 0395T, 77789, 77750, 77790, 77401, 77402, 77403, 77404, 77405, 77406, 77407, 77408, 77409, 77410, 77411, 77412, 77413, 77414, 77415, 77416, 77385, 77386, 77424, 77425, 77422, 77423, 77520, 77521, 77522, 77523, 77524, 77525, 77371, 77372, 77373, 77387, 77014, 77427, 77431, 77432, 77435, 77469, 77470
Surgery[a]	CPT – Mastectomy: 19180, 19182, 19200, 19220, 19240, 19303, 19304, 19305, 19306, 19307<br><br>CPT – Breast-conserving treatment (includes partial mastectomy): 19112, 19120, 19125, 19126, 19160, 19162, 19301, 19302
Reconstruction	CPT: 19340, 19342, 19350, 19357, 19361, 19364, 19366, 19367, 19368, 19369

[a] Excludes excisional biopsies.
CPT: Current Procedural Terminology
HCPS: Healthcare Common Procedure Coding System

© 2024 Ash R et al. *JAMA Network Open.*

eTable 3. Taxonomy Codes Used to Identify Physician Specialty

Specialty	Codes
Medical oncology	207RH0003X, 207RX0202X, 207VX0201X
Radiation oncology	2085R0001X
Surgery	2086X0206X, 208600000X, 2086S0122X, 208200000X, 208G00000X, 208C00000X

eFigure. Patient Flow Diagram

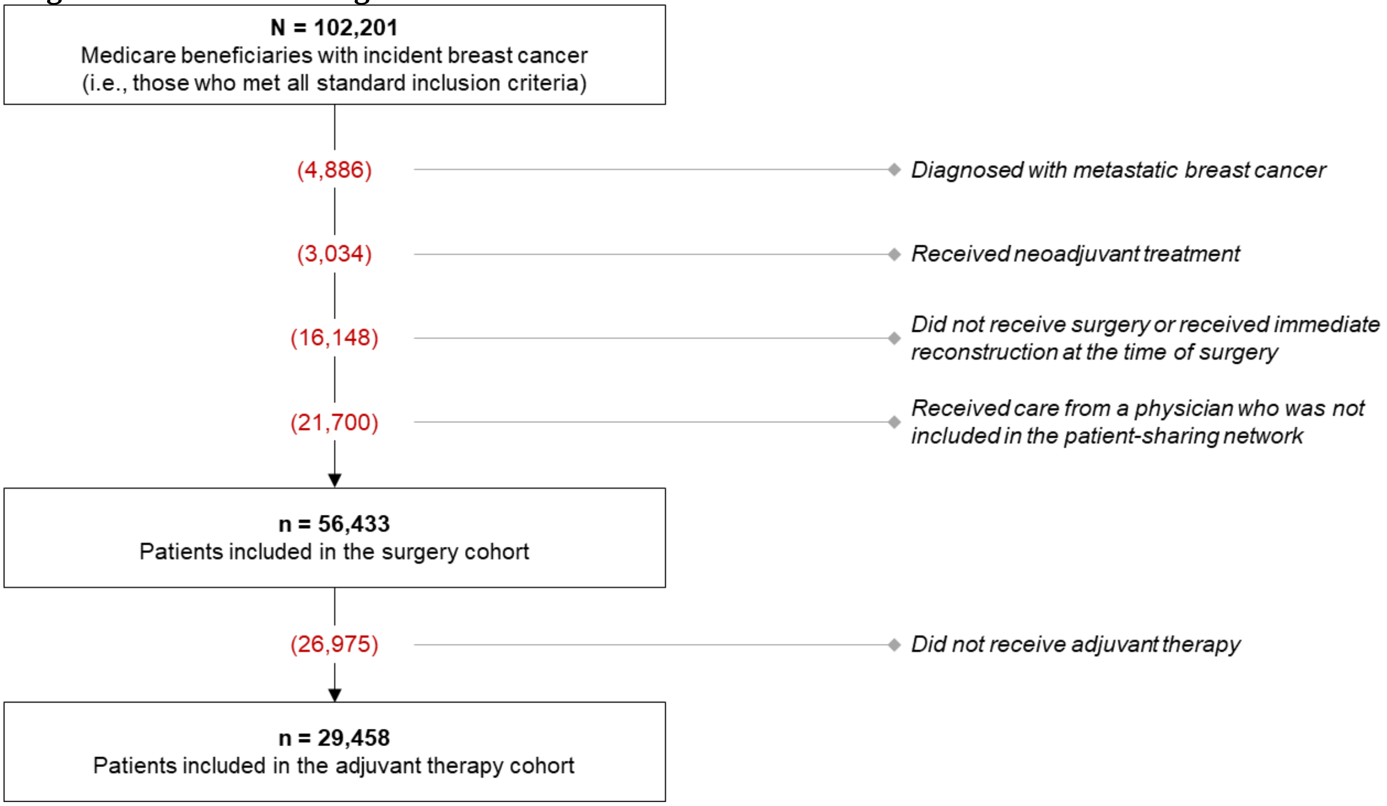

eTable 4. Bivariate Associations Between Patient Attributes of Adjuvant Therapy-Receiving Patients and Receipt of Surgery From a Linchpin Surgeon

	Adjuvant Therapy Cohort		
	Non-linchpin Surgeon	Linchpin Surgeon	*P*
	n = 24930	n = 4528	
Age at Diagnosis			
66-69	7874 (31.6%)	1385 (30.6%)	.41
70-74	8591 (34.5%)	1569 (34.7%)	
75-79	5365 (21.5%)	971 (21.4%)	
80-84	2257 (9.1%)	446 (9.8%)	
85+	843 (3.4%)	157 (3.5%)	
Race			
Asian	287 (1.2%)	45 (1.0%)	.04
Black	1466 (5.9%)	321 (7.1%)	
Hispanic	158 (0.6%)	23 (0.5%)	
North American Native	76 (0.3%)	16 (0.4%)	
Other	346 (1.4%)	59 (1.3%)	
Unknown	432 (1.7%)	67 (1.5%)	
White	22165 (88.9%)	3997 (88.3%)	
Comorbidities			
0	14286 (57.3%)	2378 (52.5%)	<.001
1	5546 (22.2%)	1068 (23.6%)	
2+	5098 (20.4%)	1082 (23.9%)	
Economic Deprivation Index			
Very Low	6884 (27.6%)	1135 (25.1%)	<.001
Low	12092 (48.5%)	2128 (47.0%)	
Medium	4119 (16.5%)	827 (18.3%)	
High	1131 (4.5%)	276 (6.1%)	
Very High	704 (2.8%)	162 (3.6%)	
NCI Affiliation			
No	21285 (85.4%)	4353 (96.1%)	<.001
Yes	3645 (14.6%)	175 (3.9%)	
Rurality			
Isolated	954 (3.8%)	233 (5.1%)	<.001
Small Rural	1208 (4.8%)	347 (7.7%)	
Large Rural	2419 (9.7%)	603 (13.3%)	
Urban	20349 (81.6%)	3345 (73.9%)	

© 2024 Ash R et al. *JAMA Network Open*.

eTable 5. Bivariate Associations Between Patient Attributes With Pre- and Postoperative Care Density Tertiles

	Surgery Cohort					Adjuvant Therapy Cohort			
	Preoperative Care Density					Postoperative Care Density			
	Sole Provider	Low	Medium	High	*P*[a]	Low	Medium	High	*P*
	n=23302	n=11044	n=11044	n=11043		n=9820	n=9819	n=9819	
Age at Diagnosis									
66-69	5354 (23.0%)	2970 (26.9%)	3061 (27.7%)	2806 (25.4%)	<.001	3116 (31.7%)	3166 (32.2%)	2977 (30.3%)	0.13
70-74	7372 (31.6%)	3483 (31.5%)	3512 (31.8%)	3637 (32.9%)		3398 (34.6%)	3351 (34.1%)	3411 (34.7%)	
75-79	5328 (22.9%)	2497 (22.6%)	2350 (21.3%)	2477 (22.4%)		2095 (21.3%)	2088 (21.3%)	2153 (21.9%)	
80-84	3244 (13.9%)	1300 (11.8%)	1291 (11.7%)	1388 (12.6%)		901 (9.2%)	870 (8.9%)	932 (9.5%)	
85+	2004 (8.6%)	794 (7.2%)	830 (7.5%)	735 (6.7%)		310 (3.2%)	344 (3.5%)	346 (3.5%)	
Race									
Asian	256 (1.1%)	162 (1.5%)	128 (1.2%)	113 (1.0%)	<.001	123 (1.3%)	112 (1.1%)	97 (1.0%)	<.001
Black	1450 (6.2%)	771 (7.0%)	644 (5.8%)	572 (5.2%)		641 (6.5%)	605 (6.2%)	541 (5.5%)	
Hispanic	116 (0.5%)	100 (0.9%)	73 (0.7%)	39 (0.4%)		86 (0.9%)	53 (0.5%)	42 (0.4%)	
North American Native	71 (0.3%)	38 (0.3%)	43 (0.4%)	23 (0.2%)		27 (0.3%)	39 (0.4%)	26 (0.3%)	
Other	320 (1.4%)	167 (1.5%)	164 (1.5%)	156 (1.4%)		132 (1.3%)	135 (1.4%)	138 (1.4%)	
Unknown	306 (1.3%)	184 (1.7%)	167 (1.5%)	170 (1.5%)		165 (1.7%)	171 (1.7%)	163 (1.7%)	
White	20783 (89.2%)	9622 (87.1%)	9825 (89.0%)	9970 (90.3%)		8646 (88.0%)	8704 (88.6%)	8812 (89.7%)	
Comorbidities									
0	12893 (55.3%)	5617 (50.9%)	5905 (53.5%)	6154 (55.7%)	<.001	5447 (55.5%)	5543 (56.5%)	5674 (57.8%)	.003
1	5333 (22.9%)	2545 (23.0%)	2503 (22.7%)	2488 (22.5%)		2204 (22.4%)	2218 (22.6%)	2192 (22.3%)	
2+	5076 (21.8%)	2882 (26.1%)	2636 (23.9%)	2401 (21.7%)		2169 (22.1%)	2058 (21.0%)	1953 (19.9%)	
Economic Deprivation Index									
Very Low	6299 (27.0%)	2873 (26.0%)	2893 (26.2%)	2960 (26.8%)	<.001	2410 (24.5%)	2665 (27.1%)	2944 (30.0%)	<.001
Low	10952 (47.0%)	5235 (47.4%)	5374 (48.7%)	5489 (49.7%)		4701 (47.9%)	4757 (48.4%)	4762 (48.5%)	
Medium	4051 (17.4%)	1905 (17.2%)	1933 (17.5%)	1778 (16.1%)		1785 (18.2%)	1664 (16.9%)	1497 (15.2%)	
High	1217 (5.2%)	615 (5.6%)	541 (4.9%)	523 (4.7%)		566 (5.8%)	446 (4.5%)	395 (4.0%)	
Very High	783 (3.4%)	416 (3.8%)	303 (2.7%)	293 (2.7%)		358 (3.6%)	287 (2.9%)	221 (2.3%)	
NCI Affiliation									
No	21048 (90.3%)	9088 (82.3%)	9664 (87.5%)	9933 (89.9%)	<.001	8233 (83.8%)	8606 (87.6%)	8799 (89.6%)	<.001
Yes	2254 (9.7%)	1956 (17.7%)	1380 (12.5%)	1110 (10.1%)		1587 (16.2%)	1213 (12.4%)	1020 (10.4%)	
Rurality									
Isolated	1044 (4.5%)	425 (3.8%)	490 (4.4%)	434 (3.9%)	<.001	474 (4.8%)	400 (4.1%)	313 (3.2%)	<.001

© 2024 Ash R et al. *JAMA Network Open.*

Small Rural	2636 (11.3%)	1084 (9.8%)	1255 (11.4%)	1001 (9.1%)		1271 (12.9%)	1023 (10.4%)	728 (7.4%)	
Large Rural	1367 (5.9%)	622 (5.6%)	598 (5.4%)	546 (4.9%)		625 (6.4%)	527 (5.4%)	403 (4.1%)	
Urban	18255 (78.3%)	8913 (80.7%)	8701 (78.8%)	9062 (82.1%)		7450 (75.9%)	7869 (80.1%)	8375 (85.3%)	

[a] *P* value calculated for all columns excluding “Sole Provider.”

© 2024 Ash R et al. *JAMA Network Open*.

eTable 6. Adjusted Associations Between Exposures and Surgical Delay

	OR (95% CI)	*P*
Main Effects		
Linchpin Status of Surgeon Yes vs. No	0.91 (0.81, 1.01)	.08
Preoperative Care Density		
Medium vs. Low	0.96 (0.89, 1.04)	.33
High vs. Low	0.58 (0.53, 0.63)	<.001
Sole-Provider vs. Low	0.35 (0.33, 0.38)	<.001
Patient-Level Characteristics		
Race		
Asian vs. White	1.15 (0.92, 1.45)	.23
Black vs. White	1.79 (1.62, 1.99)	<.001
Hispanic vs. White	1.71 (1.27, 2.29)	<.001
North American Native vs. White	1.38 (0.87, 2.20)	.17
Other vs. White	1.03 (0.83, 1.27)	.81
Unknown vs. White	0.92 (0.74, 1.15)	.48
Rurality		
Isolated vs. Urban	1.03 (0.88, 1.19)	.74
Small Rural vs. Urban	1.12 (0.98, 1.27)	.09
Large Rural vs. Urban	1.09 (0.99, 1.21)	.08
Economic Deprivation Index		
Low vs. Very Low	1.06 (1.00, 1.14)	.07
Medium vs. Very Low	1.09 (1.00, 1.19)	.05
High vs. Very Low	1.27 (1.12, 1.45)	<.001
Very High vs. Very Low	1.26 (1.08, 1.47)	.003
Age at Biopsy		
70-74 vs. 66-69	0.83 (0.77, 0.89)	<.001
75-79 vs. 66-69	0.77 (0.71, 0.83)	<.001
80-84 vs. 66-69	0.78 (0.71, 0.86)	<.001
85+ vs. 66-69	1.06 (0.95, 1.18)	.27
Comorbidities		
1 vs. 0	0.97 (0.91, 1.04)	.35
2+ vs. 0	1.02 (0.96, 1.10)	.48
NCI Affiliation: Yes vs. No	1.56 (1.40, 1.74)	<.001
Physician-Level Characteristics		
Gender: Male vs. Female	0.84 (0.78, 0.92)	<.001
Oncologist Supply		
Low vs. High	1.41 (1.26, 1.59)	<.001
Medium vs. High	1.13 (1.01, 1.26)	.04
Patient Volume		
Medium vs. Low	0.98 (0.87, 1.10)	.75
High vs. Low	1.03 (0.87, 1.22)	.72
Adjusted Within-HSA Ratio[a]		
Medium vs. Low	1.28 (1.14, 1.44)	<.001
High vs. Low	1.38 (1.17, 1.63)	<.001
Encounter-Level Characteristics		
Encounters between Biopsy and Surgery	1.12 (1.11, 1.12)	<.001

[a] Calculated as the ratio of within-HSA degree to total degree.

© 2024 Ash R et al. *JAMA Network Open.*

eTable 7. Adjusted Associations Between Exposures and Adjuvant Delay

	OR (95% CI)	*P*
Main Effects		
Linchpin Status of Surgeon Yes vs. No	1.30 (1.13, 1.49)	<.001
Postoperative Care Density		
Medium vs. Low	0.85 (0.77, 0.94)	.002
High vs. Low	0.77 (0.69, 0.87)	<.001
Patient-Level Characteristics		
Race		
Asian vs. White	0.88 (0.59, 1.30)	.51
Black vs. White	1.24 (1.05, 1.46)	.01
Hispanic vs. White	0.68 (0.40, 1.16)	.16
North American Native vs. White	1.10 (0.52, 2.32)	.79
Other vs. White	0.96 (0.68, 1.35)	.81
Unknown vs. White	0.70 (0.50, 0.98)	.04
Rurality		
Isolated vs. Urban	1.28 (1.04, 1.57)	.02
Small Rural vs. Urban	1.34 (1.12, 1.61)	.001
Large Rural vs. Urban	1.13 (0.98, 1.31)	.09
Economic Deprivation Index		
Low vs. Very Low	1.04 (0.94, 1.15)	.41
Medium vs. Very Low	1.09 (0.96, 1.24)	.18
High vs. Very Low	1.13 (0.93, 1.37)	.24
Very High vs. Very Low	1.18 (0.92, 1.50)	.19
Age at Biopsy		
70-74 vs. 66-69	0.99 (0.90, 1.09)	.85
75-79 vs. 66-69	0.98 (0.87, 1.09)	.68
80-84 vs. 66-69	1.02 (0.88, 1.19)	.77
85+ vs. 66-69	1.28 (1.03, 1.60)	.03
Comorbidities		
1 vs. 0	1.07 (0.97, 1.19)	.16
2+ vs. 0	1.21 (1.09, 1.34)	<.001
NCI Affiliation: Yes vs. No	1.67 (1.44, 1.94)	<.001
Physician-Level Characteristics		
Gender: Male vs. Female	1.08 (0.97, 1.21)	.15
Oncologist Supply		
Low vs. High	0.94 (0.80, 1.10)	.43
Medium vs. High	1.07 (0.92, 1.24)	.38
Patient Volume		
Medium vs. Low	1.06 (0.91, 1.24)	.46
High vs. Low	1.12 (0.90, 1.40)	.31
Adjusted Within-HSA Ratio[a]		
Medium vs. Low	0.90 (0.77, 1.05)	.16
High vs. Low	0.93 (0.75, 1.15)	.48
Encounter-Level Characteristics		
Encounters between Surgery & Adjuvant Therapy	2.19 (2.13, 2.25)	<.001
Treatment Type: Radiotherapy vs. Chemotherapy	2.26 (1.95, 2.62)	<.001

[a] Calculated as the ratio of within-HSA degree to total degree.

© 2024 Ash R et al. *JAMA Network Open*.

eTable 8. Adjusted Associations Between Exposures and Treatment Delay When Changing the Treatment Delay Cutoff to >90 Days, Stratified by Treatment Cohort

	Surgery Cohort		Adjuvant Therapy Cohort	
	OR (95% CI)	*P*	OR (95% CI)	*P*
Linchpin Status of Surgeon Yes vs. No	1.02 (0.88, 1.18)	.81	1.21 (0.99, 1.49)	.07
Preoperative Care Density				
Medium vs. Low	1.07 (0.96, 1.20)	.21	NA	
High vs. Low	0.64 (0.56, 0.73)	<.001	NA	
Sole-Provider vs. Low	0.33 (0.29, 0.37)	<.001	NA	
Postoperative Care Density				
Medium vs. Low	NA		0.86 (0.74, 1.00)	.05
High vs. Low	NA		0.82 (0.68, 0.98)	.03

Note: All statistical models incorporate random intercepts for physician National Provider Identifier and beneficiary HRR and control for all patient-, physician-, and encounter-level variables.

© 2024 Ash R et al. *JAMA Network Open*.
