# Supplementary material for: Surgeon and Care Team Network Measures and Timely Breast Cancer Treatment
Source: JAMA Netw Open. 2024 Aug 29;7(8):e2427451. doi: 10.1001/jamanetworkopen.2024.27451 (PMC11362867; doi:10.1001/jamanetworkopen.2024.27451)
Supplement: Supplement 2. — Data Sharing Statement [file jamanetwopen-e2427451-s002.pdf]

Data Sharing Statement

Ash. Surgeon and Care Team Network Measures and Timely Breast Cancer Treatment. *JAMA Netw Open*. Published August 29, 2024. doi:10.1001/jamanetworkopen.2024.27451

Data
Data available: No

Additional Information
Explanation for why data not available: We are prohibited from making our data publicly available due to our Data Use Agreement with ResDAC. Researchers interested in obtaining the data for their own use will need to request it directly with ResDAC.
